# Safety and efficacy of restrictive red blood cell transfusion strategy in extremely low birth weight infants: a before-after comparative study

**DOI:** 10.3389/fmed.2026.1876529

**Published:** 2026-07-30

**Authors:** Xiaozhi Xu, Xiaofeng Xu, Qiong Zhao

**Affiliations:** 1 Department of Neonatology, The First People's Hospital of Yunnan Province, The Affiliated Hospital of Kunming University of Science and Technology, Kunming, China; 2 Department of Blood Transfusion, The Affiliated Calmette Hospital of Kunming Medical University, The First People's Hospital of Kunming, Kunming, China

**Keywords:** extremely low birth weight infant, neonatal intensive care, red blood cell transfusion, restrictive transfusion, transfusion threshold

## Abstract

**Background:**

Extremely low birth weight (ELBW) infants frequently require red blood cell (RBC) transfusions, yet optimal hemoglobin thresholds remain debated. Recent evidence supports restrictive transfusion strategies, but real-world implementation data in Chinese neonatal intensive care units (NICUs) are limited. This study aimed to evaluate the safety and efficacy of implementing a restrictive RBC transfusion protocol in ELBW infants.

**Methods:**

This multicenter before-after comparative study enrolled ELBW infants (birth weight < 1,000 g) admitted to two tertiary NICUs between January 2021 and December 2025 Infants admitted during the liberal transfusion period (January 2021–December 2022 *n* = 82) were compared with those admitted after implementation of a restrictive transfusion protocol (January 2023–December 2025, *n* = 94). Liberal thresholds were hematocrit < 35% with respiratory support and < 30% without respiratory support; restrictive thresholds were hematocrit < 30% with respiratory support and < 25% without respiratory support. Primary outcomes included in-hospital mortality and composite neonatal morbidity. Secondary outcomes included the number of RBC transfusions, donor exposure, bronchopulmonary dysplasia (BPD), retinopathy of prematurity (ROP), necrotizing enterocolitis (NEC), and intraventricular hemorrhage (IVH).

**Results:**

Baseline characteristics were comparable between groups regarding gestational age (26.8 ± 1.6 vs. 26.9 ± 1.5 weeks, *P* = 0.674), birth weight (811 ± 124 vs. 804 ± 130 g, *P* = 0.713), and initial hematocrit (48.2 ± 5.1 vs. 47.8 ± 4.9%, *P* = 0.601). The restrictive group received significantly fewer RBC transfusions per infant (3.2 ± 2.1 vs. 5.8 ± 2.7, P < 0.001) and had reduced donor exposure (1.5 ± 1.2 vs. 2.9 ± 1.4 donors, *P* < 0.001). In-hospital mortality did not differ significantly between groups (9.6% vs. 11.0%, *P* = 0.955). No significant differences were observed in the incidence of BPD (38.3% vs. 42.7%, *P* = 0.662), severe ROP requiring treatment (8.5% vs. 9.8%, *P* = 0.981), NEC ≥ stage II (6.4% vs. 7.3%, *P* = 1.000), or severe IVH (grade III–IV) (10.6% vs. 12.2%, *P* = 0.931). Length of hospital stay was similar between groups (78.4 ± 24.3 vs. 81.2 ± 26.6 days, *P* = 0.465).

**Conclusion:**

Implementation of a restrictive RBC transfusion strategy in ELBW infants significantly reduced transfusion requirements and donor exposure without increasing mortality or major neonatal morbidities. These findings support the adoption of restrictive transfusion thresholds in Chinese NICUs caring for ELBW infants.

## 1 Introduction

Extremely low birth weight (ELBW) infants, defined as those with a birth weight less than 1000 grams, represent one of the most vulnerable patient populations in neonatal medicine. These infants are characterized by profound physiological immaturity and frequently develop anemia of prematurity due to a combination of inadequate erythropoiesis, shortened red blood cell lifespan, and iatrogenic blood losses from frequent laboratory sampling (1, 2). Consequently, red blood cell (RBC) transfusions remain among the most common therapeutic interventions in neonatal intensive care units (NICUs), with ELBW infants receiving an average of 5–8 transfusions during their hospital stay (3, 4).

Despite the ubiquity of RBC transfusions in neonatal practice, the optimal hemoglobin or hematocrit threshold for initiating transfusion has been a subject of ongoing debate for decades. The central question is whether a liberal transfusion strategy, which maintains higher hemoglobin levels, confers clinical benefits over a restrictive approach that tolerates lower hemoglobin concentrations (5). This debate has significant implications for clinical practice, as transfusion-related adverse effects—including transfusion-associated necrotizing enterocolitis (TANEC), transfusion-related acute lung injury (TRALI), infectious disease transmission, and donor antigen exposure—are well-documented concerns in the neonatal population (6, 7).

Two landmark randomized controlled trials have substantially advanced our understanding of transfusion thresholds in premature neonates. The Iowa trial by Bell et al. (8) and the Premature Infants in Need of Transfusion (PINT) trial (9) initially suggested that restrictive thresholds might be associated with increased risks of adverse neurodevelopmental outcomes, although the differences were small and inconsistent. More recently, the Transfusion of Prematures (TOP) trial (10) and the Effects of Transfusion Thresholds on Neurocognitive Outcomes of Extremely Low-Birth-Weight Infants (ETTNO) trial (11) provided more definitive evidence. The TOP trial, a multicenter randomized trial involving 1,824 ELBW infants, demonstrated that restrictive transfusion thresholds were non-inferior to liberal thresholds regarding the composite outcome of death or neurodevelopmental impairment at 22–26 months corrected age. Similarly, the ETTNO trial found no significant difference in the composite outcome of death or major morbidity between liberal and restrictive groups.

While these trials provide robust evidence supporting the safety of restrictive transfusion strategies, several important gaps remain. First, most evidence derives from Western populations, and data from Chinese NICUs are limited. Differences in patient demographics, clinical practices, blood bank capabilities, and healthcare infrastructure may affect the generalizability of these findings to the Chinese context (12). Second, the translation of trial-based protocols into routine clinical practice—the implementation gap—remains poorly characterized. Real-world implementation studies are essential to determine whether the safety profile observed in controlled trial settings is maintained in everyday clinical practice (13).

To address these gaps, we conducted a multicenter before-after comparative study at two tertiary hospitals evaluating the safety and efficacy of transitioning from a liberal to a restrictive RBC transfusion protocol in ELBW infants. This pragmatic study design allows assessment of the real-world impact of protocol change across different institutional settings.

## 2 Methods

### 2.1 Study design and setting

This was a multicenter, retrospective before-after comparative study conducted at the NICUs of two tertiary referral centers. The study was approved by the institutional ethics committees of both participating hospitals and was conducted in accordance with the Declaration of Helsinki. The requirement for informed consent was waived due to the retrospective nature of the study and the use of de-identified data.

### 2.2 Study population

**Figure 1:**
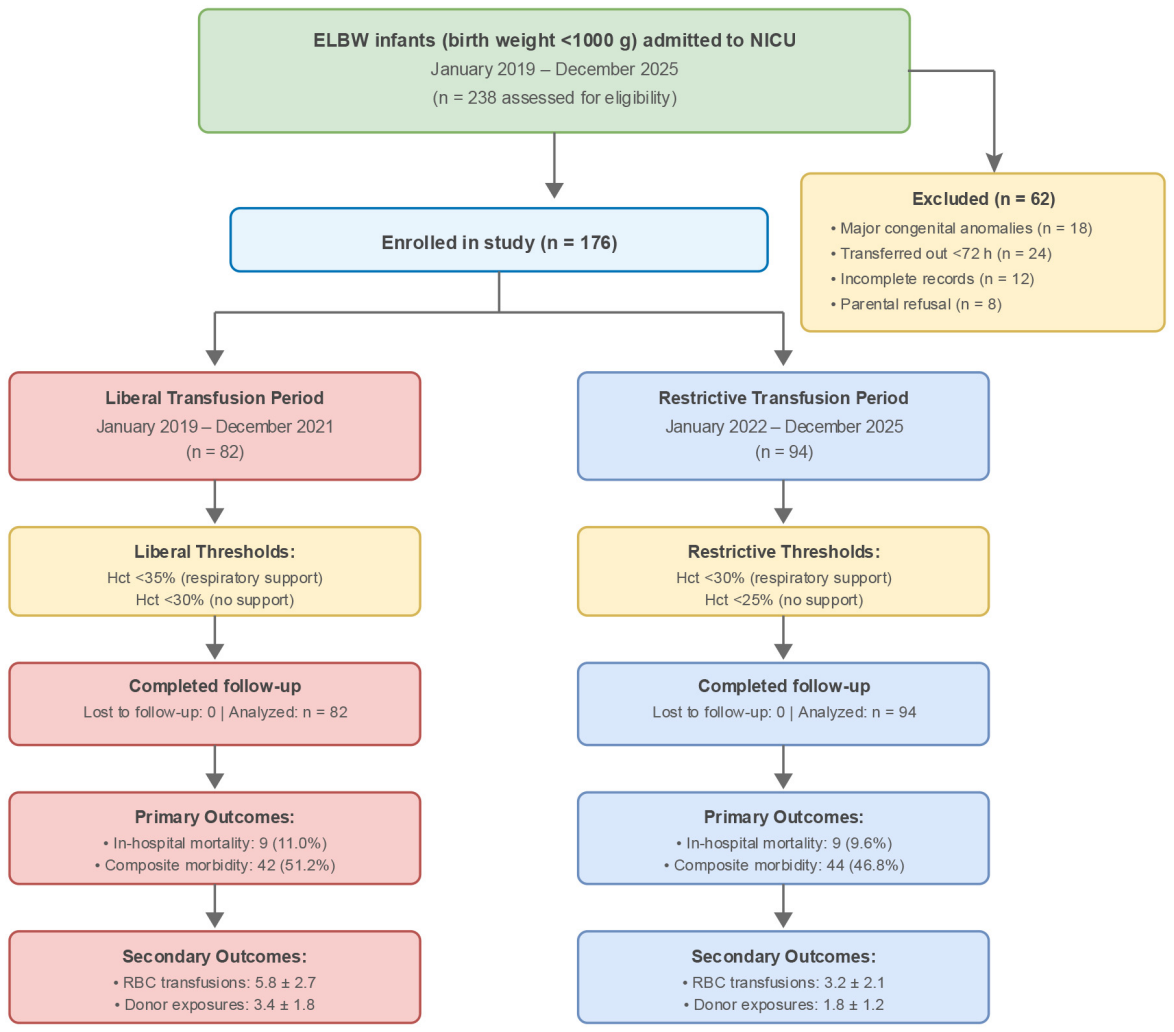
Study flow diagram. ELBW, extremely low birth weight; NICU, neonatal intensive care unit. A total of 238 infants were assessed for eligibility, and 176 were enrolled after exclusion of 62 infants.

We included all ELBW infants (birth weight < 1,000 g) admitted to two tertiary NICUs between January 1, 2021, and December 31, 2025. Exclusion criteria were: (1) major congenital anomalies incompatible with life or requiring immediate surgical intervention; (2) transfer to another facility within 72 h of admission; (3) incomplete medical records precluding outcome assessment; and (4) parental refusal of blood product transfusion for religious or personal reasons. A total of 238 ELBW infants were screened, of whom 176 met the inclusion criteria and were enrolled (Figure 1).

### 2.3 Transfusion protocol

During the liberal transfusion period (January 2021–December 2022), RBC transfusions were administered when the hematocrit fell below 35% in infants receiving respiratory support (mechanical ventilation or continuous positive airway pressure with FiO_2_ >0.25) or below 30% in infants breathing spontaneously without significant respiratory support. Beginning January 2023, a restrictive transfusion protocol was implemented based on evidence from the TOP and ETTNO trials. Under the restrictive protocol, transfusion thresholds were hematocrit < 30% with respiratory support and < 25% without respiratory support. The protocol change was implemented through a structured quality improvement initiative that included staff education, clinical pathway development, and monthly compliance monitoring.

All RBC transfusions were administered as packed red blood cells (pRBCs) at a volume of 15–20 mL/kg, infused over 3–4 h. Blood products were irradiated and leukocyte-reduced per institutional policy. When possible, divided units from a single donor were used for subsequent transfusions to minimize donor exposure. Packed RBC units supplied to both participating NICUs were leukocyte-reduced before storage and gamma-irradiated in accordance with national and institutional neonatal transfusion standards, and the sourcing and processing of blood products were not deliberately altered as part of the transfusion-threshold protocol change. Indications for other blood components (platelets and fresh-frozen plasma) followed standard institutional criteria that did not change between the two study periods.

### 2.4 Data collection

Clinical data were extracted from electronic medical records by trained research personnel using a standardized data collection form. Baseline demographic variables included gestational age, birth weight, sex, Apgar scores at 1 and 5 min, mode of delivery, multiple gestation status, antenatal steroid exposure, and maternal complications (preeclampsia, chorioamnionitis). Illness severity was quantified using the Score for Neonatal Acute Physiology with Perinatal Extension-II (SNAPPE-II) calculated within the first 12 h of admission. Recorded maternal complications were limited to preeclampsia and chorioamnionitis; maternal red-cell alloantibody status, neonatal haemolytic disease of the fetus and newborn, the timing of umbilical cord clamping and other delivery-room practices, and the cumulative volume of blood removed for laboratory testing (iatrogenic phlebotomy loss) were not systematically captured in the source records and could therefore not be analyzed. The postnatal age at which the hematocrit triggering each transfusion was measured, and the postnatal age at transfusion, were likewise not available for structured analysis in this retrospective dataset.

### 2.5 Outcome measures

The primary outcomes were in-hospital mortality and a composite of major neonatal morbidity (defined as the occurrence of one or more of the following: BPD, severe ROP requiring treatment, NEC ≥ Bell stage II, or severe IVH grade III–IV). Secondary outcomes included: (1) the number of RBC transfusions per infant; (2) the number of individual donor exposures; (3) total volume of RBC transfused (mL/kg); (4) the lowest hematocrit recorded during hospitalization; and (5) individual components of the composite morbidity outcome.

Bronchopulmonary dysplasia was defined according to the 2001 NICHD consensus definition as the need for supplemental oxygen at 36 weeks postmenstrual age (14). Severe ROP was defined as ROP requiring treatment (laser photocoagulation or intravitreal anti-VEGF injection) per the revised international classification (15). NEC was diagnosed and staged according to the modified Bell criteria (16). IVH was classified using the Papile grading system, with grades III–IV considered severe (17).

### 2.6 Statistical analysis

Continuous variables were expressed as mean ± standard deviation (SD) and compared between groups using the independent Student's *t*-test or Mann-Whitney U test, as appropriate based on normality assessment (Shapiro-Wilk test). Categorical variables were expressed as frequency and percentage and compared using the chi-square test or Fisher's exact test when expected cell counts were less than 5. Odds ratios (ORs) with 95% confidence intervals (CIs) were calculated for binary outcomes using the Restrictive group as the reference. Subgroup analyses were performed for the primary mortality outcome stratified by gestational age (< 27 vs. ≥27 weeks), birth weight (< 750 vs. ≥750 g), sex, and antenatal steroid exposure. A two-sided *P*-value < 0.05 was considered statistically significant. All analyses were performed using Python 3.9 with SciPy 1.11 and pandas 2.0 statistical libraries.

## 3 Results

### 3.1 Study population and baseline characteristics

During the 5-year study period, 238 ELBW infants were admitted to the two participating NICUs. After applying exclusion criteria, 176 infants were enrolled: 82 in the liberal transfusion group (January 2021–December 2022) and 94 in the restrictive transfusion group (January 2023–December 2025). The patient flow is depicted in Figure 1.

Baseline demographic and clinical characteristics were well-balanced between the two groups (Table 1). The mean gestational age was 26.8 ± 1.6 weeks in the liberal group and 26.9 ± 1.5 weeks in the restrictive group (*P* = 0.674). Mean birth weight was 811 ± 124 g and 804 ± 130 g, respectively (*P* = 0.713). Initial hematocrit values were similar (48.2 ± 5.1% vs. 47.8 ± 4.9%, *P* = 0.601). No significant differences were observed in sex distribution, Apgar scores, delivery mode, multiple gestation rates, antenatal steroid exposure, maternal complications, illness severity scores, surfactant use, or PDA requiring treatment (all *P* > 0.05).

**Table 1:** Baseline demographic and clinical characteristics of the study population.

Variable	Liberal (*n* = 82)	Restrictive (*n* = 94)	*P* value
Gestational age (weeks)	26.8 ± 1.6	26.9 ± 1.5	0.674
Birth weight (g)	811 ± 124	804 ± 130	0.713
Initial hematocrit (%)	48.2 ± 5.1	47.8 ± 4.9	0.601
Male sex, *n* (%)	43 (52.4)	47 (50.0)	0.864
Apgar score at 1 min	4.5 ± 1.9	4.6 ± 1.7	0.915
Apgar score at 5 min	7.0 ± 1.4	7.0 ± 1.4	0.776
Cesarean delivery, *n* (%)	51 (62.2)	60 (63.8)	0.946
Antenatal steroids, *n* (%)	67 (81.7)	78 (83.0)	0.982
Multiple gestation, *n* (%)	18 (22.0)	22 (23.4)	0.961
Small for GA, *n* (%)	15 (18.3)	18 (19.1)	1.000
Maternal preeclampsia, *n* (%)	16 (19.5)	20 (21.3)	0.919
Chorioamnionitis, *n* (%)	12 (14.6)	15 (16.0)	0.973
SNAPPE-II score	30.4 ± 11.9	29.9 ± 11.4	0.758
Surfactant use, *n* (%)	70 (85.4)	81 (86.2)	1.000
PDA treatment, *n* (%)	29 (35.4)	31 (33.0)	0.862

Data are presented as mean ± SD or *n* (%). *P* values from student's *t*-test (continuous) or chi-square/fisher's exact test (categorical). GA, gestational age; PDA, patent ductus arteriosus; SNAPPE-II, Score for Neonatal Acute Physiology with Perinatal Extension-II.

### 3.2 Transfusion-related outcomes

**Figure 2:**
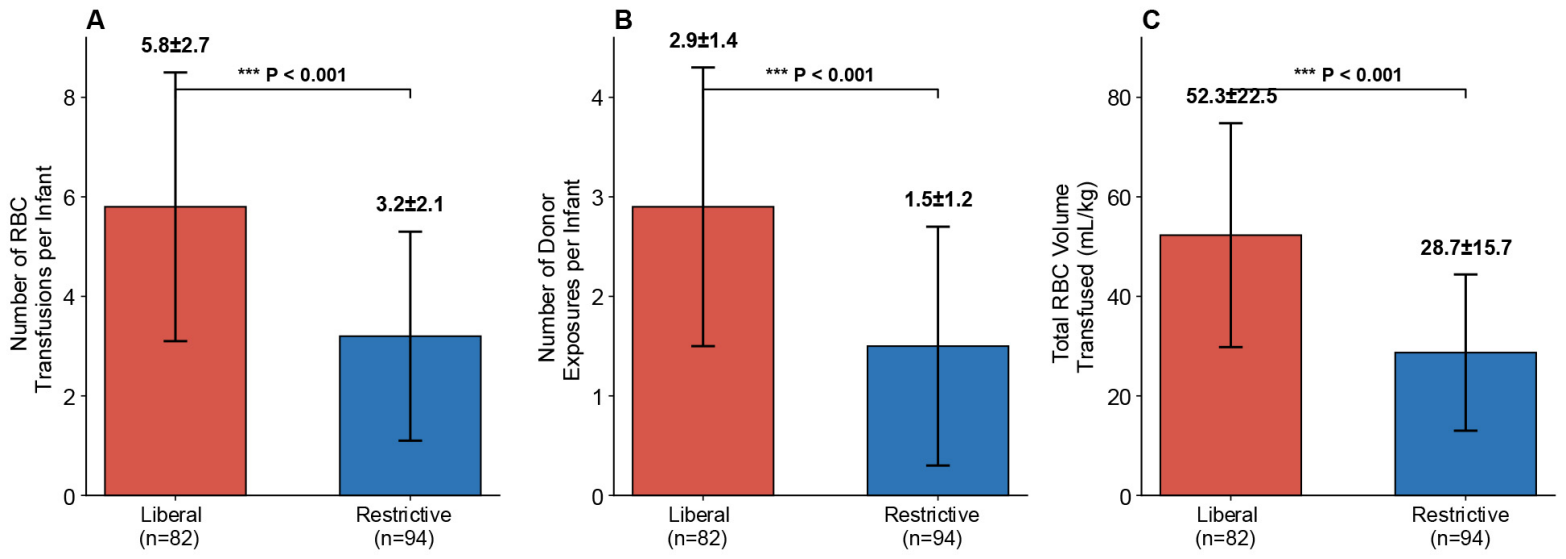
Comparison of transfusion-related outcomes between the liberal (red) and restrictive (blue) groups. **(A)** Number of RBC transfusions per infant. **(B)** Number of donor exposures per infant. **(C)** Total RBC volume transfused (mL/kg). Data are presented as mean ± SD with individual data points. ***P < 0.001.

**Figure 3:**
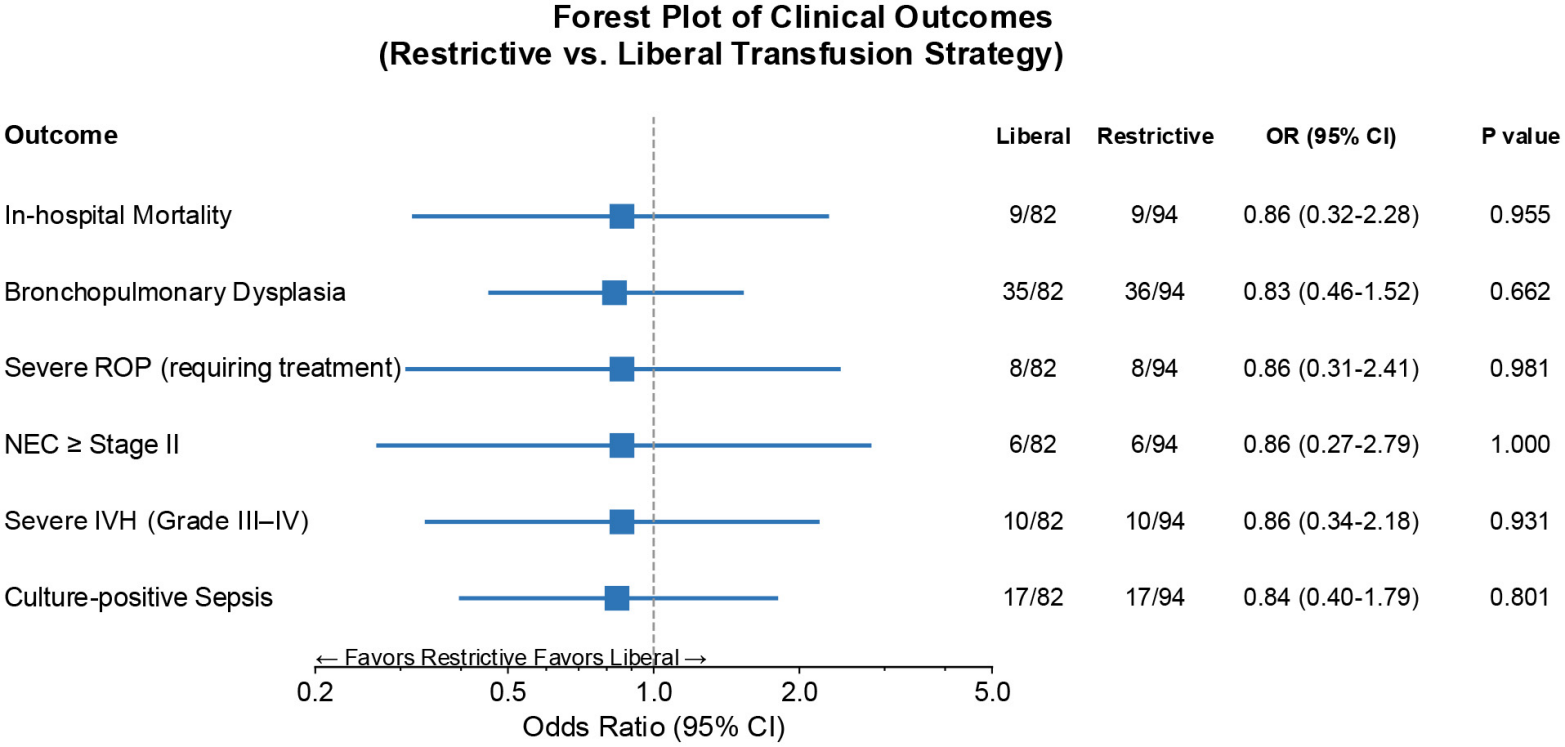
Box plots comparing hematocrit values between groups. **(A)** Initial hematocrit at admission showing no significant difference (*P* = 0.601). **(B)** Lowest hematocrit during hospitalization showing significantly lower values in the restrictive group (*P* < 0.001), as expected from the lower transfusion thresholds. Individual data points are overlaid.

Implementation of the restrictive transfusion protocol resulted in significant reductions in all transfusion-related parameters (Table 2, Figure 2). Infants in the restrictive group received significantly fewer RBC transfusions per infant compared with the liberal group (3.2 ± 2.1 vs. 5.8 ± 2.7, *P* < 0.001), representing a 44.8% reduction. Donor exposure was similarly reduced (1.5 ± 1.2 vs. 2.9 ± 1.4 exposures, *P* < 0.001), a 48.3% reduction. The total volume of RBC transfused was markedly lower in the restrictive group (28.7 ± 15.7 vs. 52.3 ± 22.5 ml/kg, *P* < 0.001). As expected, the lowest recorded hematocrit during hospitalization was significantly lower in the restrictive group (24.8 ± 3.8% vs. 28.5 ± 4.2%, *P* < 0.001; Figure 3).

**Table 2:** Primary and secondary outcomes.

Outcome	Liberal (*n* = 82)	Restrictive (*n* = 94)	Difference/OR (95% CI)	*P* value
*Transfusion outcomes*				
RBC transfusions/infant	5.8 ± 2.7	3.2 ± 2.1	−2.6	< 0.001
Donor exposures/infant	2.9 ± 1.4	1.5 ± 1.2	−1.4	< 0.001
Total RBC volume (mL/kg)	52.3 ± 22.5	28.7 ± 15.7	−23.6	< 0.001
Lowest hematocrit (%)	28.5 ± 4.2	24.8 ± 3.8	−3.7	< 0.001
*Primary outcomes*				
In-hospital mortality	9 (11.0)	9 (9.6)	0.86 (0.32–2.28)	0.955
Composite morbidity	42 (51.2)	44 (46.8)	0.84 (0.46–1.52)	0.563
*Secondary outcomes*				
BPD	35 (42.7)	36 (38.3)	0.83 (0.46–1.52)	0.662
Severe ROP	8 (9.8)	8 (8.5)	0.86 (0.31–2.41)	0.981
NEC ≥ Stage II	6 (7.3)	6 (6.4)	0.86 (0.27–2.79)	1.000
Severe IVH (III–IV)	10 (12.2)	10 (10.6)	0.86 (0.34–2.18)	0.931
Culture-positive sepsis	17 (20.7)	17 (18.1)	0.84 (0.40–1.79)	0.801
*Other outcomes*				
Mech. ventilation (days)	14.5 ± 8.2	13.8 ± 7.6	−0.7	0.531
TPN duration (days)	26.3 ± 9.5	25.2 ± 8.6	−1.1	0.411
Time to full feeds (days)	24.8 ± 7.5	23.5 ± 7.0	−1.3	0.239
Length of stay (days)	81.2 ± 26.6	78.4 ± 24.3	−2.8	0.465
Weight at discharge (g)	2,350 ± 420	2,311 ± 399	−39.4	0.525

Data are presented as mean ± SD or *n* (%). Continuous variables compared using student's *t*-test; categorical variables compared using chi-square or fisher's exact test. OR, odds ratio; CI, confidence interval; BPD, bronchopulmonary dysplasia; ROP, retinopathy of prematurity; NEC, necrotizing enterocolitis; IVH, intraventricular hemorrhage; TPN, total parenteral nutrition.

### 3.3 Primary outcomes

In-hospital mortality was 9.6% (9/94) in the restrictive group compared with 11.0% (9/82) in the liberal group, with no statistically significant difference (OR 0.86, 95% CI: 0.32–2.28, *P* = 0.955). The composite neonatal morbidity outcome occurred in 46.8% (44/94) of restrictive group infants vs. 51.2% (42/82) of liberal group infants (OR 0.84, 95% CI: 0.46–1.52, *P* = 0.563). These results indicate that the restrictive strategy did not increase the risk of mortality or major morbidity.

### 3.4 Secondary outcomes

**Figure 4:**
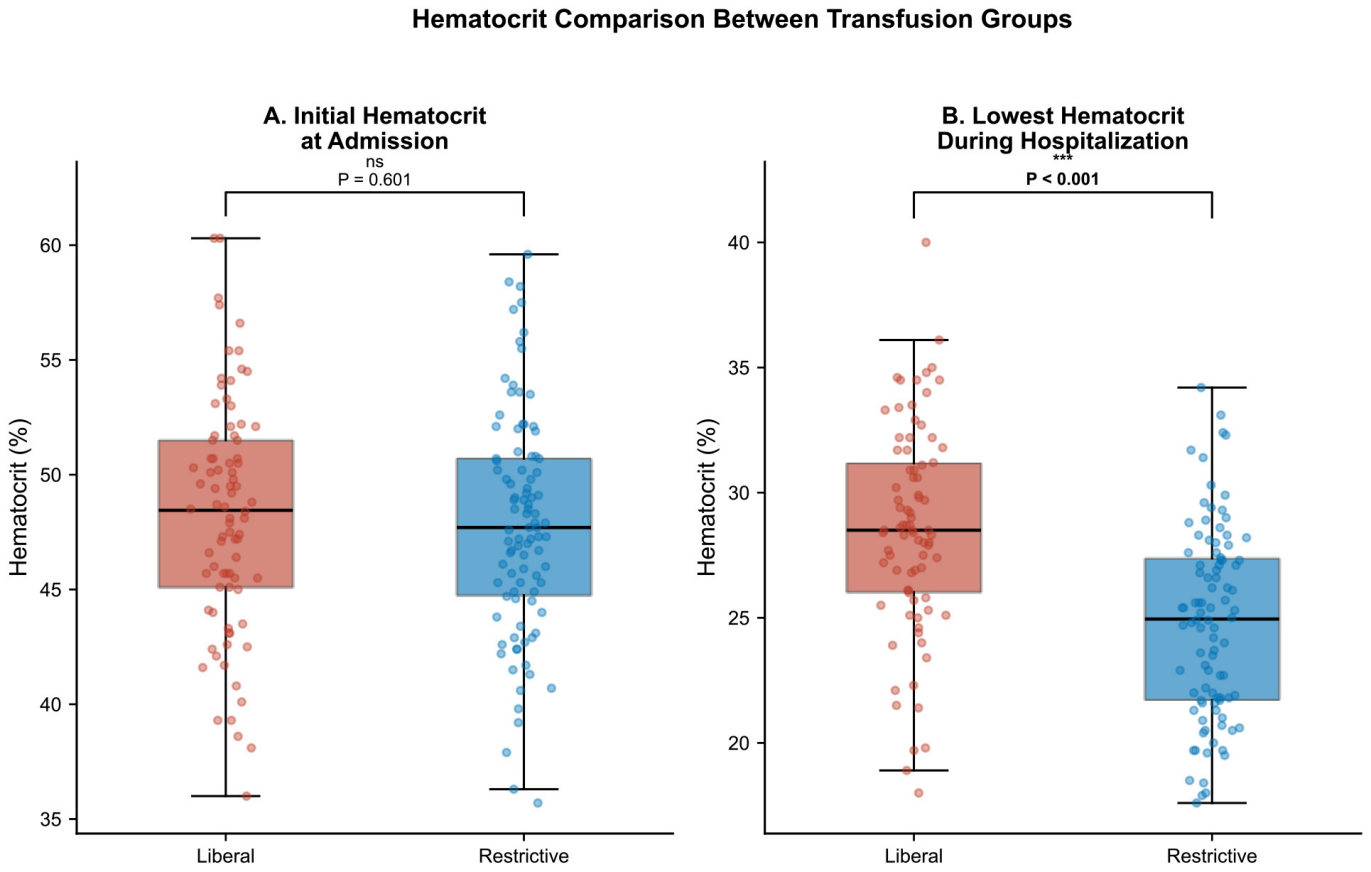
Forest plot of clinical outcomes comparing the restrictive vs. liberal transfusion strategy. Odds ratios with 95% confidence intervals are shown. Values less than 1.0 favor the restrictive strategy. BPD, bronchopulmonary dysplasia; ROP, retinopathy of prematurity; NEC, necrotizing enterocolitis; IVH, intraventricular hemorrhage.

Individual components of the composite morbidity outcome showed no significant differences between groups (Table 2, Figure 4). BPD was diagnosed in 38.3% (36/94) of restrictive group infants vs. 42.7% (35/82) of liberal group infants (OR 0.83, 95% CI: 0.46–1.52, *P* = 0.662). Severe ROP requiring treatment occurred in 8.5% (8/94) vs. 9.8% (8/82) of infants (OR 0.86, 95% CI: 0.31–2.41, *P* = 0.981). NEC ≥ stage II was observed in 6.4% (6/94) vs. 7.3% (6/82) (OR 0.86, 95% CI: 0.27–2.79, *P* = fdata1.000). Severe IVH (grade III–IV) occurred in 10.6% (10/94) vs. 12.2% (10/82) (OR 0.86, 95% CI: 0.34–2.18, *P* = 0.931). Culture-positive sepsis rates were also comparable (18.1% vs. 20.7%, *P* = 0.801). Acute transfusion reactions—including transfusion-related acute lung injury and transfusion-associated circulatory overload—and transfusion-transmitted infections were not systematically adjudicated as discrete endpoints in this retrospective dataset; transfusion-associated necrotizing enterocolitis is subsumed within the NEC outcome reported above (6.4% vs. 7.3%, *P* = 1.000).

Other clinical parameters did not differ significantly between groups. Duration of mechanical ventilation (13.8 ± 7.6 vs. 14.5 ± 8.2 days, *P* = 0.531), total parenteral nutrition duration (25.2 ± 8.6 vs. 26.3 ± 9.5 days, *P* = 0.411), time to full enteral feeds (23.5 ± 7.0 vs. 24.8 ± 7.5 days, *P* = 0.239), and length of hospital stay (78.4 ± 24.3 vs. 81.2 ± 26.6 days, *P* = 0.465) were similar between groups.

### 3.5 Subgroup analysis

**Figure 5:**
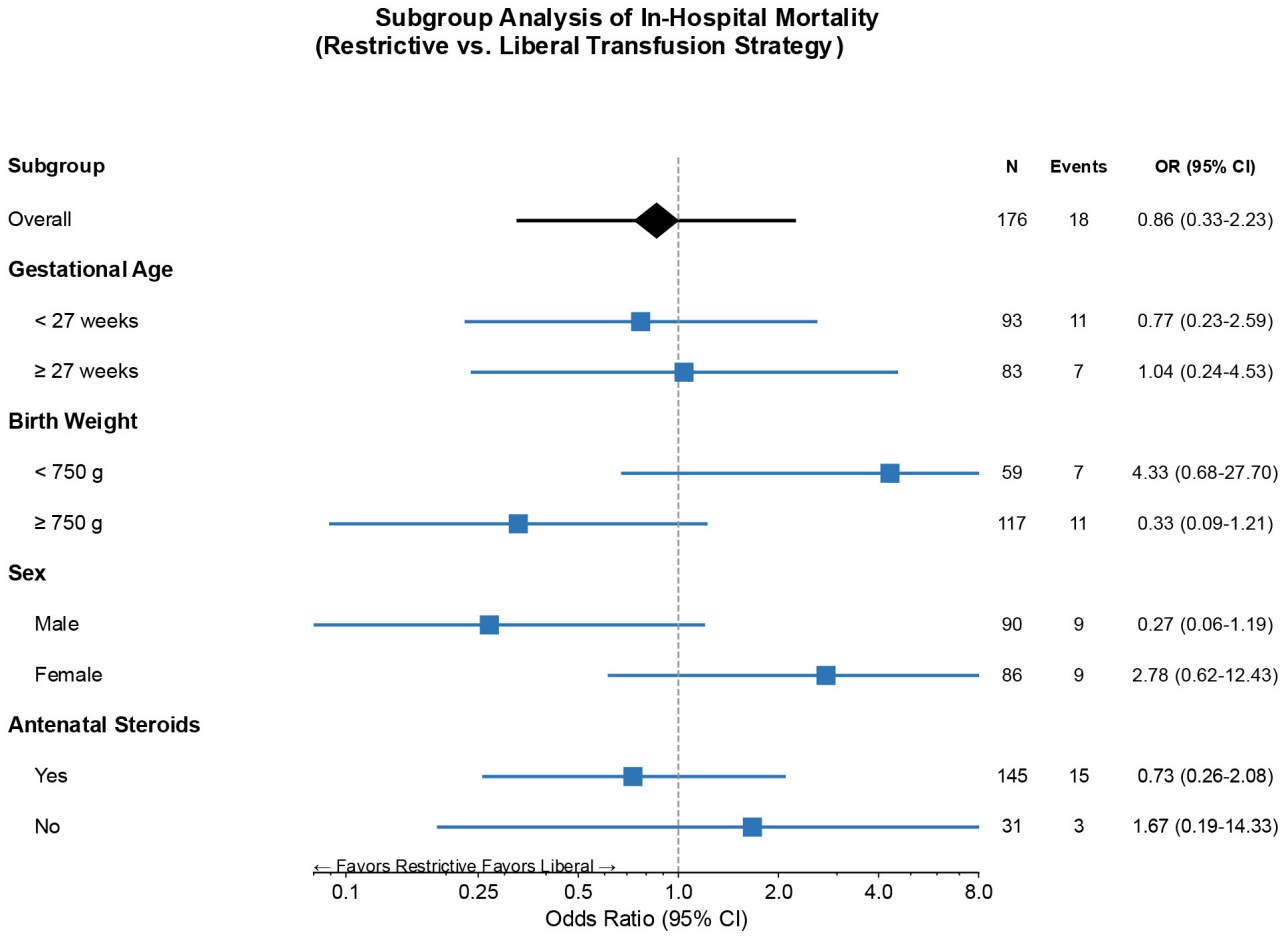
Subgroup analysis of in-hospital mortality (restrictive vs. liberal). Odds ratios with 95% confidence intervals are shown for each subgroup. The dashed vertical line represents an OR of 1.0 (no difference). No significant interactions were detected between any subgroup variable and the transfusion strategy.

Subgroup analyses of the primary mortality outcome stratified by gestational age, birth weight, sex, and antenatal steroid exposure revealed consistent results across all subgroups (Figure 5). The point estimates for odds ratios consistently favored or were neutral for the restrictive strategy, with all 95% confidence intervals crossing 1.0. No significant interaction was detected between any subgroup variable and transfusion strategy, suggesting that the safety of the restrictive approach was maintained regardless of these baseline characteristics.

## 4 Discussion

This multicenter before-after comparative study demonstrates that implementation of a restrictive RBC transfusion strategy in ELBW infants significantly reduced transfusion burden—including the number of transfusions, donor exposures, and total transfused volume—without increasing in-hospital mortality or major neonatal morbidities. These findings are consistent with the results of the landmark TOP and ETTNO trials and provide important real-world evidence supporting the adoption of restrictive transfusion thresholds in Chinese NICUs. Our real-world findings are concordant with contemporary international guidance: the 2024 clinical practice guideline of Deschmann et al. (24) recommends restrictive hemoglobin thresholds for very preterm neonates, and the consensus transfusion guidelines reported by Gilmore et al. (23) for a large neonatal intensive care network similarly endorse standardized, predominantly restrictive practice.

The magnitude of transfusion reduction observed in our study is clinically meaningful. The restrictive strategy was associated with a 44.8% reduction in transfusion episodes and a 48.3% reduction in donor exposure. These findings are consistent with previous implementation studies reporting reductions of 40–70% following the adoption of restrictive protocols (18, 19). The reduction in donor exposure is particularly noteworthy, as each additional donor exposure carries cumulative risks of alloimmunization, transfusion-transmitted infections, and immune modulation (20). In our practice, the use of divided units from single donors further amplified the reduction in donor exposure beyond what would be expected from reduced transfusion frequency alone.

Regarding safety outcomes, our finding of comparable mortality rates between groups (9.6% vs. 11.0%, *P* = 0.955) is reassuring and consistent with the TOP trial, which reported 16.2% mortality in the liberal group vs. 16.0% in the restrictive group (10). The slightly lower overall mortality in our study population may reflect differences in gestational age distribution, center-specific practices, or temporal improvements in neonatal care over our study period. Importantly, subgroup analyses demonstrated that the safety of the restrictive strategy was consistent across clinically relevant subgroups, including those defined by gestational age, birth weight, sex, and antenatal steroid exposure.

The similar rates of BPD (38.3% vs. 42.7%), severe ROP (8.5% vs. 9.8%), NEC (6.4% vs. 7.3%), and severe IVH (10.6% vs. 12.2%) between groups are of particular clinical importance. Theoretical concerns have been raised that lower hematocrit levels in restrictive strategies might impair oxygen delivery to developing organs, potentially exacerbating conditions such as BPD and ROP (21). Our data, along with those from the TOP and ETTNO trials, provide evidence against this hypothesis in the clinical context of the hematocrit thresholds studied. The lowest hematocrit observed in the restrictive group (24.8 ± 3.8%) remained within the range generally considered acceptable for stable ELBW infants without significant cardiorespiratory compromise (22). These null differences in mortality and major morbidity, accompanied by a clear reduction in transfusion exposure, reproduce the conclusions of the most recent systematic reviews and meta-analyses in very low birth weight infants, including the updated Cochrane review by Andersen et al. (25) and the meta-analysis by Wang et al. (26), both of which reported no increase in mortality, neurodevelopmental impairment, or other serious morbidity with restrictive thresholds alongside modest reductions in transfusion burden.

Our study has several strengths. First, it provides real-world implementation data from Chinese NICUs, addressing an important evidence gap given the predominance of Western populations in existing trials. Second, the multicenter design across two tertiary hospitals enhances generalizability compared with single-center studies. Third, the before-after design with a clear temporal separation of transfusion protocols minimizes contamination between groups. Fourth, the comprehensive assessment of both safety and efficacy outcomes provides a balanced evaluation of the protocol change. Fifth, baseline characteristics were well-matched between groups, reducing the risk of confounding.

Several limitations should be acknowledged. First, the retrospective before-after design is inherently susceptible to temporal confounding, as improvements in overall neonatal care over the study period may have influenced outcomes independent of the transfusion protocol change. Second, although this multicenter study included two tertiary hospitals, both were located in the same city, which may limit generalizability to other regions of China. Third, the sample size, while adequate for our primary analysis, limits statistical power for subgroup analyses and rare outcomes. Fourth, long-term neurodevelopmental outcomes, which represent the most clinically important endpoint, were not assessed in this study and should be evaluated in future prospective investigations. Fifth, the before-after design does not allow for randomization, and unmeasured confounders may exist despite the balanced baseline characteristics. Furthermore, the unequal observation windows between the two groups (liberal group: 2 years, January 2021–December 2022; restrictive group: 3 years, January 2023–December 2025) may introduce temporal confounding bias, as advances in neonatal care over time could independently contribute to outcome differences beyond the transfusion protocol change itself. In addition, several variables of potential relevance were not available in this retrospective dataset and could not be incorporated into the analysis: maternal red-cell alloantibody status and neonatal haemolytic disease of the fetus and newborn; the anticoagulant–preservative solution and detailed processing of individual blood units; the timing of umbilical cord clamping and other delivery-room practices that influence hematocrit at birth; the cumulative volume of iatrogenic phlebotomy loss; and the postnatal age at which the hematocrit determining each transfusion was measured and at which transfusion was administered. Because systemic and pulmonary blood-flow distribution and postnatal hematocrit evolve rapidly during the fetal-to-neonatal transition, the postnatal timing of these measurements and exposures may influence outcomes; the absence of these data is acknowledged as a limitation. Acute transfusion-associated events were likewise not systematically adjudicated. Finally, although the transfusion-threshold protocol was the principal planned change between the two periods, we cannot fully exclude concurrent secular changes in delivery-room, respiratory, or nutritional practice, or in unit staffing and leadership, that may have contributed to outcomes independently of the transfusion strategy.

Our findings have important implications for clinical practice and policy in China. The demonstrated safety and efficacy of restrictive transfusion thresholds support the development of national guidelines incorporating these thresholds for ELBW infants. Implementation should be accompanied by structured education programs, clinical pathway standardization, and compliance monitoring systems, as were employed in our study. Future multicenter prospective studies with neurodevelopmental follow-up are needed to further validate these findings in the Chinese neonatal population.

## 5 Conclusion

Implementation of a restrictive RBC transfusion strategy in ELBW infants significantly reduced the number of transfusions by 44.8%, donor exposures by 48.3%, and total transfused volume by 45.1%, without increasing in-hospital mortality (9.6% vs. 11.0%, *P* = 0.955) or the incidence of major neonatal morbidities including BPD, severe ROP, NEC, and severe IVH. Subgroup analyses confirmed the consistency of these findings across clinically relevant patient subgroups. These results provide real-world evidence supporting the adoption of restrictive transfusion thresholds (hematocrit < 30% with respiratory support, < 25% without) in Chinese NICUs caring for ELBW infants. Future large-scale multicenter prospective studies with long-term neurodevelopmental follow-up are warranted to further validate these findings across diverse Chinese populations.

## Data Availability

The original contributions presented in the study are included in the article/supplementary material, further inquiries can be directed to the corresponding author.
